# *HR* Gene Variants Identified in Mexican Patients with Alopecia Areata

**DOI:** 10.3390/cimb45040194

**Published:** 2023-04-03

**Authors:** Andrés Ortiz-Ramírez, María Cristina Hernández-Jiménez, Iliana Berenice Guardiola-Avila, Erick de Jesús De Luna-Santillana, Amanda Alejandra Oliva-Hernández, María Lourdes Altamirano-García, Karina Janett Juárez-Rendón

**Affiliations:** 1 Centro de Biotecnología Genómica, Instituto Politécnico Nacional, Blvd. del Maestro s/n. Esq. Elías Piña, Col. Narciso Mendoza, Reynosa 88710, Mexico; 2 Unidad Académica Multidisciplinaria Reynosa Aztlán, Universidad Autónoma de Tamaulipas, Calle 16 s/n y Lago de Chapala, Col. Aztlán, Reynosa 88740, Mexico; 3 Department of Immunology and Microbiology, School of Medicine, University of Texas Rio Grande Valley (UTRGV), 5300 L. St., McAllen, TX 78504, USA; 4 Centro Dermatológico Brunnenhalt de Reynosa, Vicente Guerrero 805 altos, Zona Centro, Reynosa 88500, Mexico

**Keywords:** alopecia areata, *HR* gene variants, Mexican population, bioinformatics

## Abstract

Alopecia Areata (AA) is a multifactorial, dermatological disease characterized by non-scarring hair loss. Alterations in candidate genes, such as *HR* (Hairless), could represent a risk factor for its development. The aim of this study was to search for and analyze variants in exons 3, 15 and 17 of the *HR* gene in Mexican patients with AA. A total of 30 samples from both AA patients and healthy donors were analyzed in this study. Exons were amplified and sequenced using the Sanger method. Descriptive statistics and χ2 tests were used in the analysis of clinical–demographic characteristics and the comparison of allelic/genotypical frequencies between groups, respectively. The effect on protein function for the non-synonymous variants was determined with three bioinformatics servers. Three gene variants were identified in the *HR* gene of the evaluated patients. The benign polymorphism c.1010G > A p.(Gly337Asp) (rs12675375) had been previously reported, whereas the variants c.750G > A p.(Gln250Gln) and c.3215T > A (Val1072AGlu) have not been described in other world populations. Both non-synonymous variants proved to be significant (*p* ≤ 0.05). The variant c.3215T > A p.(Val1072Glu) is of particular interest due to its deleterious effect on the structure and function of the protein; therefore, it could be considered a risk factor for the development of AA.

## 1. Introduction

Alopecia areata (AA) is a dermatological disease characterized by non-scarring hair loss that has been associated with high rates of depression and anxiety. Especially in women, self-esteem is seriously affected [1,2]. The lifetime risk of developing the disease is nearly 2% in the general population [3], with an estimated prevalence of 0.1–6.9% depending on the evaluated population [4]. Both genders, all ethnicities and age groups can be affected; however, this condition usually occurs between the second and third decade of life [5]. In Mexico, the prevalence of AA oscillates between 0.2 and 3.8% and is observed between 20 and 40 years [6].

Clinically, some patients show the first signs of AA in patched form (PAA) localized to the scalp, arms, legs, eyelashes, beards, or eyebrows, although they may experience a rapid progression to total alopecia (TA), during which total loss of facial and scalp hair occurs; or even present universal alopecia (UA), involving the loss of all body hair [7].

AA is of multifactorial origin. Previous studies have proposed that the immune mediated inhibition of the hair follicle, genetic and/or environmental components could trigger its development; however, the precise mechanisms by which this occurs are largely unknown [8]. Concerning the genetics of AA, it has been suggested that this condition does not follow a common Mendelian pattern attributed to a single gene locus [9]; on the contrary, all the genes involved in the hair follicle cycle (HFC) and immune and inflammatory responses could be suitable candidates during their study [10,11]. The Hairless (*HR*) gene is a particularly important example. It is located at locus 8p21.3, consists of 17 exons and encodes the HR protein, which weighs 130 kDa and is composed of 1189 amino acids [12]. Research in both murine (mouse) and human models has indicated that this gene is a nuclear transcription factor essential in skin maintenance and hair follicle development [13,14], which acts as an apoptosis regulator during the catagen phase and as a keratinocyte inhibitor through its repression function over the Vitamin D receptor, although its role is poorly understood [15]. Likewise, the *HR* gene is expressed in brain and other tissues, and, interestingly, about 200 mutations have been identified in various types of cancer, including prostate, breast, lung and uterine, suggesting that it is also important in the growth and survival of cancer cells [12].

The human has become a suitable model for the study of AA given the cases described. In the *HR* gene, some variants associated with the development of congenital alopecia have been identified [16]. Studies in patients of Pakistani and Italian origin identified mutations in exons 3 [17], 15 [18], and 17 [19], respectively. However, it is well known that differences could be observed in the populations analyzed. To the best of our knowledge, there is no evidence in this regard for the Mexican population. Therefore, this initial study includes the search and analysis of variants in exons 3, 15 and 17 of the *HR* gene in Mexican AA patients.

## 2. Materials and Methods

### 2.1. Sample

A total of 30 samples were analyzed, of which 15 were obtained from AA patients and 15 from healthy donors among the general population. The participants included both men and women aged over 18 years of Mexican nationality. They all signed an informed consent form (004/2018/CEI). The procedures were performed according to the ethical principles and norms established in the declaration of Helsinki. The study is descriptive, transversal, and comparative.

### 2.2. Molecular and Bioinformatics Analysis

To perform the molecular analysis, exons 3, 15, and 17 of the *HR* gene were amplified by PCR using the primers shown in Table 1, and then sequenced by the Sanger method (ABI 3130; Applied Biosystems, Foster City, CA, USA). The identified gene variants were confirmed three times. The bioinformatic tools, Sorting Intolerant From Tolerant (SIFT), Polymorphism Phenotyping-2 (PolyPhen-2), and Mutation Taster, available at: https://sift.bii.a-star.edu.sg (accessed on 29 January 2023), http://genetics.bwh.harvard.edu/pph2/ (accessed on 2 February 2023) and http://www.mutationtaster.org (accessed on 3 February 2023), respectively, were used to determine the functional impact of the proteins coded in the non-synonymous gene variants.

### 2.3. Statistical Analysis

Descriptive statistical analyses were used in the evaluation of clinical–demographic variables (mean and frequency). The number of genotypes was calculated by the simple count method. Allelic and genotypic frequencies between patients and healthy donors were determined with Arlequin v3.0 software and compared using a χ2 test with SPSS v23 software (SPSS Inc., Chicago, IL, USA). A *p* ≤ 0.05 was considered significant.

## 3. Results

### 3.1. Clinical and Demographic Data

This study, the first of its kind in a Mexican population, includes the analysis of fifteen AA patients (i.e., PAA, TA, or UA). The mean age of the participants was 39 years, with an average age at diagnosis of 21 years. The majority of the patients showed symptoms before 20 years or in the range of 21–40 years of age (n = 8; 53.3%). Most patients were male (n = 9; 60%), of which 11 (73.3%) had a familiar history of AA, with PAA as the more common clinical type (n = 13; 86.7%).

### 3.2. Molecular and Bioinformatics Analysis

**Figure 1:**
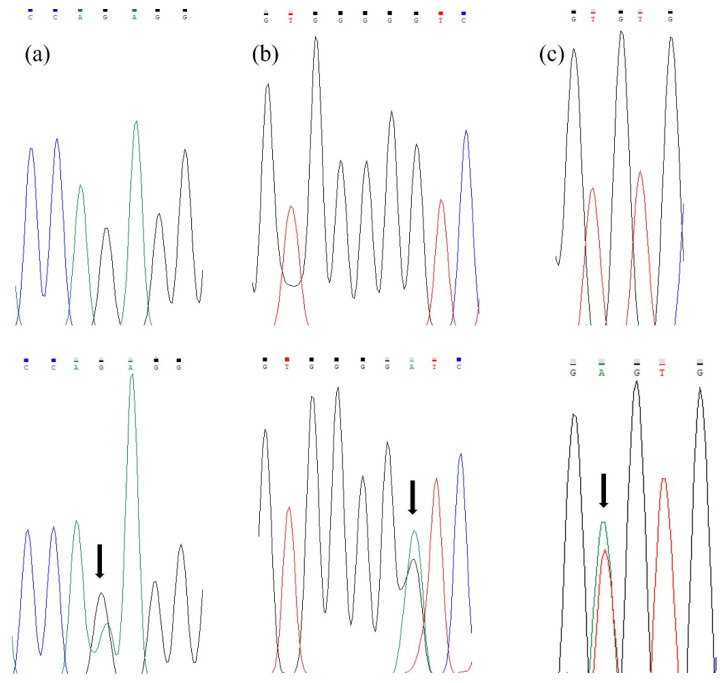
Electropherograms showing both the wild-type homozygous state of the *HR* gene and its heterozygous state. Illustrated in this figure are the unreported synonymous transition c.750G > A p.(Gln250Gln) (**a**), the non-synonymous transition c.1010G > A p.(Gly337Asp) (previously reported as polymorphism rs12675375) (**b**), and the previously unreported non-synonymous transition c.3215T > A p.(Val1072Glu) (**c**).

During the molecular analysis, a total of three gene variants were identified (Figure 1), two of which were detected in exon 3 in a heterozygous state. The first of these variants was the unreported synonymous transition c.750G > A p.(Gln250Gln), observed in one patient (7%) and in two healthy donors (13%). The second was the non-synonymous transition c.1010G > A p.(Gly337Asp), previously described as a polymorphism (rs12675375), that was observed in eight patients (53%) and one healthy donor (7%). The third gene variant was detected in exon 17 and was classified as the non-synonymous transversion, c.3215T > A p.(Val1072Glu), which has not been reported in other populations. In the present study, this gene change was discovered in a heterozygous state only in the analyzed patients (n = 7; 47%). No gene variants were identified in exon 15. The allelic and genotypic frequencies are shown in Table 2. The population was in Hardy–Weinberg equilibrium. The gene variant c.750G > A observed in exon 3 was not determined to be significant, whereas the variants c.1010G > A and c.3215T > A that were identified in exons 3 and 17, respectively, did have statistical significance *p* ≤ 0.05 (Table 2). The bioinformatic analysis performed with the three proposed tools predicted a benign effect for the non-synonymous variant c.1010G > A p.(Gly337Asp), whereas the variant c.3215T > A (Val1072AGlu) had a clear deleterious effect over the normal function of the protein (Table 3).

## 4. Discussion

The *HR* gene has been proposed as a candidate for AA especially because it plays a role in the development of the hair follicle [20]. However, previous studies in different world populations have described some gene variants mainly associated with UA [18,19]; its role in patients with PAA and TA needs to be determined. There are no previous reports in the Mexican population; therefore, the results provided in this report should be of great interest in the study of this dermatological illness. Despite the sample size, we identified a total of three variants in the coding region of the *HR* gene, of which the unreported synonymous transition c.750G > A p.(Gln250Gln) and the already described non-synonymous transition c.1010G > A p.(Gly337Asp) (rs12675375) were detected in exon 3. Although no amino acid changes were observed in the synonymous variant, previous studies have reported that such alterations often affect the stability and translation efficiency of the coded protein [21]. The previously reported polymorphism, rs12675375, did not affect protein function (benign) despite resulting in an amino acid change, as determined by the tools SIFT, Polyphen 2, and Mutation Taster (Table 3), and in concurrence with other databases widely used in genetic studies such as NCBI (ClinVar = benign) [22] and the ACMG (The American College of Medical Genetics and Genomics) classification (likely benign) (https://varsome.com/variant/hg38/rs12675375?%29= (accessed on 6 March 2023)), thus demonstrating that not all amino acid changes have a negative effect, regardless of the clinical type, given that in our study this variant was mainly identified in patients with PAA (n = 8; 53%), whereas in previous reports it was observed in patients with congenital UA. In addition, the allele frequency analysis for this polymorphism was consistent with those reported in the GnomAD (27% vs. 31%, respectively) [22], a database characterized by aggregating both exome and genome sequencing results from large-scale sequencing projects.

On the other hand, in exon 17, we identified the non-synonymous transversion c.3215T > A p.(Val1072Glu), which has not been previously reported in other world populations; interestingly, this variant was only discovered in patients with PAA (n = 5; 33.3%) or with UA (n = 2, 13.3%) and was completely absent in the healthy donors included in this study. Furthermore, the change was located within the C-terminal Jumonji C (JmjC)-like domain (residues 946 to 1156), in the human HR protein which has been described to display histone demethylase (HDM) activity, and literature data indicate that HR D1020N and V1056M mutations from patients with atrichia with papular lesions (APL) markedly decrease the HDM activity compared to the wild type, thus any alteration in this domain is relevant in the hair cycle and skin maintenance [12]. Other studies have reported that the HR protein can inhibit the expression of several nuclear receptors including thyroid hormone, retinoic acid, and vitamin D involved in the regulation of the HFC; therefore, a structural change in the protein could affect its regulatory capacity over these receptors resulting in HFC disbalance [23,24]. In addition to this, the statistical analysis showed that these results are significant (*p* ≤ 0.05), and the bioinformatics analysis predicted a deleterious effect on the encoded protein, supporting the idea that this variant could represent a risk factor for the development of AA.

The results obtained in this initial study revealed that the populations could behave differently. In the Mexican population, we did not identify any of the gene variants previously reported by Ahmed et al., 2013 [17], Ahmad et al., 1998 [18], and Nucara et al., 2011 [19], in exons 3, 15 and 17, respectively, although we identified a previously reported polymorphism and two new gene variants in exons 3 and 17; therefore, it is important to consider that regardless of the sample size, ethnic differences should be considered in the search of gene variants associated with AA.

Concerning the clinical–demographic characteristics, it was determined that the age of onset was before 20 years of age or in the range of 21 to 40 years (n = 8; 53.3%), which is consistent with previous reports stating that the first symptoms of AA appear between the first and second decades of life [25], with peak incidence (52%) between 20 and 40 years [26]. Additionally, the mean age of diagnosis was 21 years, while in other populations, it is reported to be between 31 and 33 years [5,27]. The predominant gender in this population was male (n = 9; 60%). Interestingly, the patients with more severe forms of UA were also male, similar to previous reports, although the gender may vary according to the studied population [11]. Most of our patients presented PAA (n = 13; 86.7%), which corresponds to the most common type of alopecia (88%) [26]. Moreover, 73.3% of the affected patients (n = 11) had a family history of AA, while other studies have described that genetics play an important role in 10–50% of cases [28].

The study of AA in the Mexican population is very important given the scarce reports of genes associated with the disease. However, our work has certain limitations. On the one hand, it is necessary both to search for gene variants in larger patient populations and to analyze other ethnic groups in order to determine whether our results correspond to those reported by other populations or whether the variants identified are specific to the Mexican population. On the other hand, it is important to perform in vivo studies that support the deleterious effect on the protein determined in this study.

## 5. Conclusions

We identified three *HR* gene variants in Mexican patients with AA. The gene variant c.3215T > A p.(Val1072Glu), which has not been reported previously in other world populations, could be a risk factor for the development of AA, although additional studies in larger patient populations are needed in order to support the results observed.

## Figures and Tables

**Table 1:** Primers for the amplification of exons 3, 15 and 17 of the *HR* gene.

Exon	Fragment Size	Forward	Reverse	Amplification Programs
3	835 pb	5′-AGTCAGCTGA AGCGTAACAC-3′	5′-CCTTACCTTT CTGCTCATCA-3′	95 °C for 1 min (1 cycle), followed by 29 cycles at 95 °C for 30 s, 60 °C for 30 s and 72 °C for 1 min; finally, 1 cycle at 72 °C for 7 min.
15	299 pb	5′-AGTGCCAGGA TTACAGGCGT-3′	5′-CTGAGGAGGAAAGAGCGCTC-3′	95 °C for 1 min (1 cycle), followed by 32 cycles at 95 °C for 30 s, 60 °C for 30 s and 72 °C for 1 min; finally, 1 cycle at 72 °C for 7 min.
17	297 pb	5′-CTGGAAAGTC CATGCCCCAT-3′	5′-GTCGCTTCTG CCATCCTGAT-3′	95 °C for 1 min (1 cycle), followed by 30 cycles at 95 °C for 30 s, 59 °C for 30 s and 72 °C for 1 min; finally, 1 cycle at 72 °C for 7 min.

**Table 2:** *HR* gene variant frequencies.

Exon	Variant	Genotype	Genotype Frequency (AA Patients %)	Genotype Frequency (Healthy Individuals %)	*p* Value	Allele	Allele Frequency (AA Patients %)	Allele Frequency (Healthy Individuals %)	*p* Value
3	c.750G > A p.(Gln250Gln)	GG	14 (93)	13 (87)	0.543	G	29 (97)	28 (93)	0.554
		GA	1 (7)	2 (13)		A	1 (3)	2 (7)	
		AA	0 (0)	0 (0)					
		Total	15 (100)	15 (100)			30 (100)	30 (100)	
	c.1010G > A p.(Gly337Asp) rs12675375	GG	7 (47)	14 (93)	0.005 *	G	22 (73)	29 (97)	0.011 *
		GA	8 (53)	1 (7)		A	8 (27)	1 (3)	
		AA	0 (0)	0 (0)					
		Total	15 (100)	15 (100)			30 (100)	30 (100)	
17	c.3215T > A p.(Val1072Glu)	TT	8 (53)	15 (100)	0.003 *	T	23 (77)	30 (0)	0.005 *
		TA	7 (47)	0 (0)		A	7 (23)	0 (0)	
		AA	0 (0)	0 (0)					
		Total	15 (100)	15 (100)			30 (100)	30 (100)	

* A *p* ≤ 0.05 was considered significant.

**Table 3:** Bioinformatic analysis for non-synonymous variants.

Variant	Bioinformatic Server					
	Polyphen2		Mutation Taster		SIFT	
	Effect	Score	Effect	Probability	Effect	Score
c.1010G > A p.(Gly337Asp)	Benign	0.005	Polymorphism (Probably harmless).	0.99	Tolerated	0.48
c.3215T > A p.(Val1072Glu)	Probably damaging	0.998	Disease causing	0.99	Damaging	0.00

## Data Availability

All data used in this paper are available in the article.
